# Craniofacial morphometric analysis of individuals with X-linked hypohidrotic ectodermal dysplasia

**DOI:** 10.1002/mgg3.84

**Published:** 2014-05-20

**Authors:** Alice F Goodwin, Jacinda R Larson, Kyle B Jones, Denise K Liberton, Maya Landan, Zhifeng Wang, Anne Boekelheide, Margaret Langham, Vagan Mushegyan, Snehlata Oberoi, Rosalie Brao, Timothy Wen, Ramsey Johnson, Kenneth Huttner, Dorothy K Grange, Richard A Spritz, Benedikt Hallgrímsson, Andrew H Jheon, Ophir D Klein

**Affiliations:** 1 Program in Craniofacial and Mesenchymal Biology, University of California San FranciscoSan Francisco, CA; 2 Department of Cell Biology & Anatomy, McCaig Bone and Joint Institute, University of CalgaryCalgary, Alberta, Canada; 3 Canadian Institutes of Health Research Training Program in Genetics, Child Development and Health, Alberta Children's Hospital Research Institute for Child and Maternal Health, University of CalgaryCalgary, Alberta, Canada; 4 Center for Craniofacial Anomalies, Department of Orofacial Sciences, University of California San FranciscoSan Francisco, CA; 5 Edimer Pharmaceuticals IncCambridge, MA; 6 Washington University in St. LouisSt. Louis, MO; 7 Human Medical Genetics and Genomics Program, University of Colorado School of MedicineAurora, CO; 8 Alberta Children's Hospital Foundation, Institute for Child and Maternal Health, University of CalgaryCalgary, Alberta, Canada; 9 Institute for Human Genetics and Department of Pediatrics, University of California San FranciscoSan Francisco, CA

**Keywords:** 3D imaging, craniofacial development, ectodysplasin, geometric morphometrics, X-linked hypohidrotic ectodermal dysplasia

## Abstract

Hypohidrotic ectodermal dysplasia (HED) is the most prevalent type of ectodermal dysplasia (ED). ED is an umbrella term for a group of syndromes characterized by missing or malformed ectodermal structures, including skin, hair, sweat glands, and teeth. The X-linked recessive (XL), autosomal recessive (AR), and autosomal dominant (AD) types of HED are caused by mutations in the genes encoding ectodysplasin (*EDA1*), EDA receptor (*EDAR*), or EDAR-associated death domain (*EDARADD*). Patients with HED have a distinctive facial appearance, yet a quantitative analysis of the HED craniofacial phenotype using advanced three-dimensional (3D) technologies has not been reported. In this study, we characterized craniofacial morphology in subjects with X-linked hypohidrotic ectodermal dysplasia (XLHED) by use of 3D imaging and geometric morphometrics (GM), a technique that uses defined landmarks to quantify size and shape in complex craniofacial morphologies. We found that the XLHED craniofacial phenotype differed significantly from controls. Patients had a smaller and shorter face with a proportionally longer chin and midface, prominent midfacial hypoplasia, a more protrusive chin and mandible, a narrower and more pointed nose, shorter philtrum, a narrower mouth, and a fuller and more rounded lower lip. Our findings refine the phenotype of XLHED and may be useful both for clinical diagnosis of XLHED and to extend understanding of the role of EDA in craniofacial development.

## Introduction

Ectodermal dysplasia (ED) encompasses more than 150 clinically distinct syndromes, all of which exhibit defects in the morphogenesis of ectodermal structures, including skin, hair, sweat glands, and teeth (Clauss et al. 2008). Hypohidrotic ectodermal dysplasia (HED) is the most prevalent type of ED and can be inherited in an X-linked (XL) recessive, autosomal recessive (AR), or autosomal dominant (AD) manner. X-linked hypohidrotic ectodermal dysplasia (XLHED) (OMIM #305100) is caused by mutations in *EDA1*, encoding ectodysplasin (Mikkola 2009). AR-HED and AD-HED are caused by mutations in *EDAR*, encoding the EDA receptor, or *EDARADD*, encoding EDAR-associated death domain (EDARADD) (Mikkola 2009). In humans, *EDA1* is expressed in multiple tissues including various epithelia, neuroectoderm, thymus, and bone during embryonic and fetal development and in adulthood (Montonen et al. 1998). The clinical features of HED include sparse hair and eyebrows, wrinkled and dry skin, missing and malformed teeth, hypoplasia of sweat, sebaceous, meibomian, lacrimal, and mammary glands, and severe hypohidrosis (Mikkola 2009). Mice with spontaneous mutations in *Eda* (tabby), *Edar* (downless), or *Edaradd* (crinkled) exhibit abnormal phenotypes similar to humans with HED, including missing teeth, teeth with abnormal cusp morphology, absent hair types, and missing sweat glands (Courtney et al. 2005).

Previous studies of individuals with HED using clinical dysmorphologic and cephalometric evaluations have identified the following craniofacial characteristics in these patients: maxillary hypoplasia, mandibular prognathism, facial concavity, frontal prominence, and depressed nasal bridge (Clauss et al. 2008). In this study, we extended the craniofacial phenotype of HED using three-dimensional (3D) imaging and geometric morphometric (GM) analysis, which applies multivariate statistical techniques to defined landmarks to precisely quantify shape and size variation in complex morphologies (Zelditch et al. 2004). By contrast, cephalometric techniques capture only dimensional differences rather than changes in overall morphology or shape. 3D morphometric analysis has great potential in clinical diagnosis of syndromes associated with craniofacial dysmorphologies and has been applied to a number of syndromes, including Noonan syndrome, fragile X syndrome, and others (Hammond et al. 2004; Heulens et al. 2012). Morphometric analysis has been utilized to identify subtle changes in craniofacial features that are difficult to observe by clinical examination, and this can help to define phenotypically distinct subgroups within a syndrome (Hammond et al. 2012a) and to discover genotype–phenotype correlations (Bhuiyan et al. 2006; Hammond et al. 2012b). Here, in a cohort of 23 male subjects with XLHED, we characterize facial morphology using 3D GM analysis.

## Material and Methods

### Study subject demographics

This study received Institutional Review Board approval. Patients were enrolled in the study at the University of California, San Francisco in May 2011 or the National Foundation for Ectodermal Dysplasias (NFED) Family Conference in Houston, TX in July 2013. All study subjects, or their legal guardians if subjects were under 18 years of age, provided written informed consent prior to participation in the study. A total of 59 healthy male control subjects with no family history of XLHED and 23 male case subjects with a genetically proven diagnosis of XLHED participated in the study. *EDA1* mutations are listed in Table 1. The 23 XLHED subjects consisted of three pairs of brothers and 17 unrelated individuals. Control subjects were all unrelated. The age range of the XLHED cohort was 4–29 years (mean 15.83 years), and ethnic backgrounds included Caucasian (*n* = 19), Hispanic (*n* = 2), and African American (*n* = 2). Ages of the control subjects ranged from 4 to 31 years (mean 12.22 years), with all controls having Caucasian ethnic background (*n* = 59). The age mismatch between the two groups is due to a larger number of younger subjects in the control group, which were included to better show phenotypic variation in the control sample. When cases and controls are matched one-to-one, the mean age difference between the two groups is very small (control mean age = 16.00 years, XLHED mean age = 15.82).

**Table 1:** Gene mutations in our cohort of 23 XLHED individuals

*EDA1* gene sequence	Mutation type	Region in ectodysplasin affected
Exon 01 R156H	Missense	Transmembrane
Exon 01 164T>A (Leu55Gln)[Table-fn tf1-1]	Missense	Transmembrane
Exon 02 463C>T (Arg155Cys)[Table-fn tf1-1]	Missense	Furin
Exon 02 467G>A (Arg156His)	Missense	Furin
Exon 02 C332Y	Missense	TNF
Exon 02 novel R384S	Missense	Furin
Exon 03 463C>T (Arg155Cys)	Missense	Furin
Exons 03-08 del	Deletion	TNF
Exon 04 553_588 del36 (185-196 del (GlyXY)X4)	Deletion, in frame	Collagen
Exon 05 546_581 del36	Deletion	Furin
Exon 06 766C>T (Gln256X)*	Nonsense	TNF
Exon 07 794A>G (Asp265Gly)	Missense	TNF
Exon 07 822G>T (Trp274Cys)	Missense	TNF
Exon 07 822 delG	Deletion, truncating	TNF
Exon 07 895G>A (Gly299Ser)	Missense	TNF
Exon 07 809 delT (Val270GlyfsX10)	Deletion	TNF
Exon 08 ?_925 1176_? del	Deletion	TNF
Exon 08 1070G>C (Arg357Pro)	Missense	TNF
Exon 08 1087A>G (Lys363Glu)	Missense	TNF
E67X mutation in *EDA1* gene	Nonsense	Extracellular

* Denotes mutation of brother pair.

### 3D imaging and landmarking

**Figure 1:**
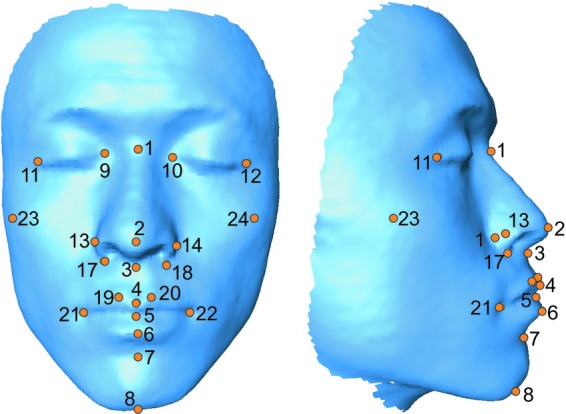
3D landmarks collected from digitized 3D facial photographs. These correspond to landmarks in Table 2.

3D facial images were created using the 3D Capturor II camera system (InSpeck, Montréal, Canada), utilizing white light 3D photogrammetry to create a 3D surface map in ∼0.4 sec with a 640 × 480-mm field of view. Following digital reconstruction of the 3D images, 3D landmarks were determined using MeshLab software (Cignoni et al. 2008). Figure 1 and Table 2 show the 24 discrete anatomical landmarks that were utilized to define and measure the shape of the craniofacial and midfacial complexes. The landmarking protocol included the use of type 1 and type 2 landmarks (Bookstein 1997).

**Table 2:** Facial landmarks utilized in morphometric analysis

Number	Name	Landmark description
1 (M)	Naison	Midline point where the frontal and nasal bones contact (nasofrontal suture).
2 (M)	Pronasale	Midline point marking the maximum protrusion of the nasal tip.
3 (M)	Subnasale	Midline point marking the junction between the inferior border of the nasal septum and the cutaneous upper lip. Apex of the nasolabial angle.
4 (M)	Labiale Superius	Midline point of the vermilion border of the upper lip, at the base of the philtrum.
5 (M)	Stomion	Midpoint of the labial fissure.
6 (M)	Labiale Inferius	Midline point of the vermilion border of the lower lip.
7 (M)	Sublabiale	Midpoint along the inferior margin of the cutaneous lower lip.
8 (M)	Gnathion	Midline point on the inferior border of the mandible.
9 (R/L)	Endocanthion	Apex of the angle formed at the inner corner of the palpebral fissure where the upper and lower eyelids meet.
11 (R/L)	Exocanthion	Apex of the angle formed at the outer corner of the palpebral fissure where the upper and lower eyelids meet.
13 (R/L)	Alare	Most lateral point on the nasal ala.
15 (R/L)	Alare Curvature Point	Most posterolateral point on the alar cartilage, located within the crease formed by the union of the alar cartilage and the skin of the cheek.
17 (R/L)	Subalare	Point located at the lower margin of the nasal ala, where the cartilage inserts in the cutaneous upper lip.
19 (R/L)	Christa Philtri	Point marking the lateral crest of the philtrum of the upper lip.
21 (R/L)	Chelion	Point marking the lateral extent of the labial fissure.
23 (R/L)	Zygion	Most prominent portion of the zygomatic arch.

### Statistical shape analyses

**Figure 2:**
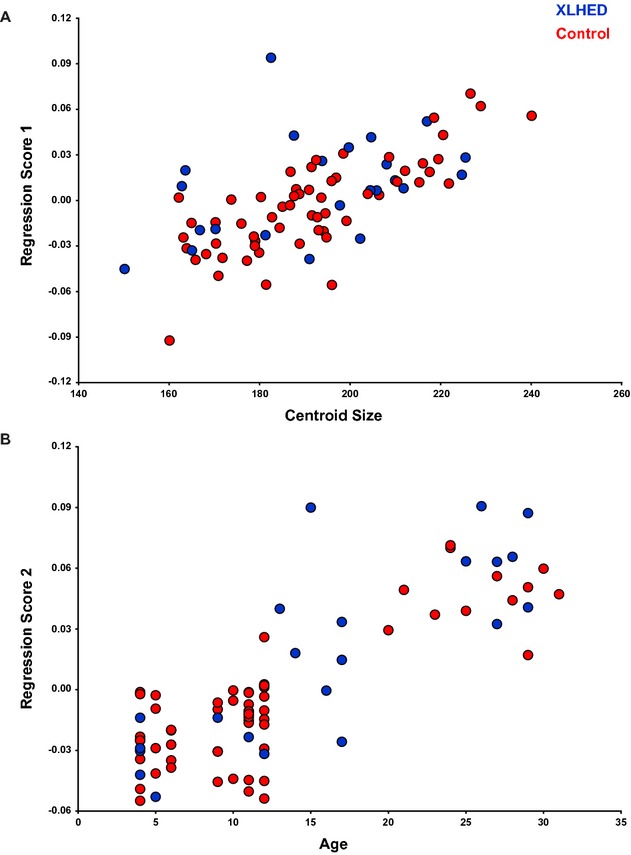
Multivariate pooled within-group regression of shape on centroid size (A) and age (B). Please see text for discussion.

The shape analyses tested the null hypothesis that XLHED subjects did not have statistically different facial shape compared to control subjects. We used GM methods, based on Procrustes superimposition, to quantify the shape and size of XLHED and control subjects (Bookstein 1997). Procrustes coordinates were calculated using the Procrustes generalized least squares superimposition method in MorphoJ software (Klingenberg 2011) which removes isometric scaling, rotational, and translational data from the landmark coordinates (Rohlf and Slice 1990). The symmetric component of each coordinate was extracted from the landmark coordinates, and the resulting coordinates were used as shape variables in subsequent analyses. As a measure of size, for each subject we computed centroid size, which is the square root of the sum of the squared distances of each landmark coordinate from the centroid, or the mean *x*, *y*, *z* coordinate (Bookstein 1997). In virtually all complex morphological traits, a substantial component of the variation in shape is directly correlated with size (Klingenberg 1998; Hallgrimsson et al. 2009). This variation, or allometry, can confound comparisons in which there is both a size and a shape effect. Even if the groups do not differ in size, removing the allometric component of variation will sharpen the focus on the morphological differences between the groups. Here, we removed both size- (static allometry) and age-(ontogenetic allometry) related variation from the coordinates using pooled within-group multivariate regression of shape on centroid size and age in years (Fig. 2A and B). The residuals of this regression were used in subsequent statistical shape analyses.

To examine the effects of the mutation on size, we regressed centroid size against age, and performed a *t*-test on the residuals. To visualize shape variation within the entire sample, we performed principal components analysis (PCA). PCA is a multivariate data reduction technique that summarizes patterns of variation and covariation by extracting independent and orthogonal axes of covariation, termed principal components (PCs), from a multivariate dataset. Each PC describes an axis of shape variation that explains a progressively smaller proportion of the total variation in the data (Zelditch et al. 2004). The shape variation described by each PC can be visualized as a 3D morphing of facial shape. 3D morphings of shape axes were generated by warping the 3D surface of an unaffected control, using the thin-plate spline procedure in the Landmark software (Wiley et al. 2005). We additionally compared the eigenvector lengths of each landmark associated with PCs 1–3 to identify which landmarks were strongly associated with each PC.

To visualize shape variation among XLHED and control individuals, we performed canonical variates analysis (CVA). CVA is similar to PCA in that canonical variates (CVs) are a linear combination of the original variables, constrained to be mutually orthogonal (Zelditch et al. 2004), which scales the shape variation to the pooled within-group covariance matrix to maximize among-group shape variation (Zelditch et al. 2004). In addition to testing for differences between affected XLHED and control groups, we also performed CVA to test for differences based on ethnicity (Caucasian, African American, and Hispanic), type of *EDA1* mutation (nonsense, missense, or deletion), and region of the EDA protein affected (tumor necrosis factor [TNF], furin, or transmembrane domain).

As there were three pairs of siblings present in the subject sample, one sibling from each pair was removed before performing the PCA and CVA so as not to artificially reduce variation in the sample due to shared facial similarity among related individuals. The eigenvalues for the PCs and CVs were exported, and the PC and CV scores for these three individuals were imputed into this shape space by summing the eigenvectors across the regression residuals for each individual using the statistical software R. Therefore, while they are depicted in the plots and analyses, there was no loss of power due to relatedness as it was based on the variation in the unrelated sample only.

## Results

### Facial shape of XLHED individuals differs from controls

**Figure 3:**
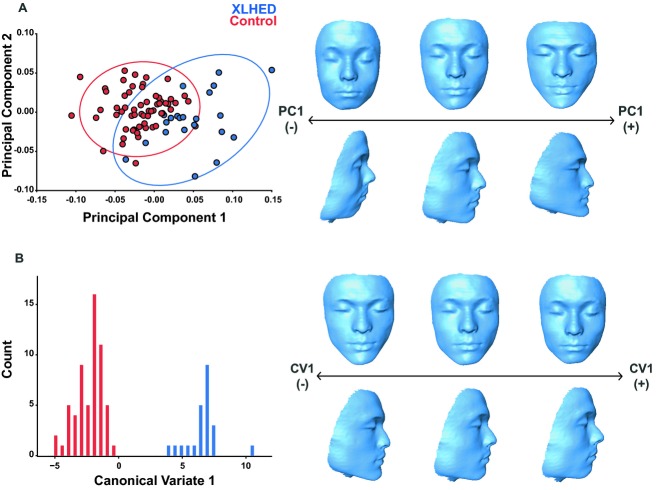
Multivariate shape analyses of craniofacial features of XLHED subjects compared to controls. (A) PC1 versus PC2, showing shape distribution of XLHED and control individuals. Ellipses correspond to 95% confidence intervals. Thin-plate spline warps illustrate the shape changes in PC1, corresponding to the observed zero, positive, and negative extreme values. (B) Canonical variate (CV) analysis histogram showing shape distribution of XLHED and control individuals. Thin-plate spline warps illustrate the shape changes in CV1, corresponding to the observed zero, positive, and negative extreme values.

Pooled within-group multivariate regression of shape on centroid size and age revealed that 22.91% of shape variation within the dataset was due to static and ontogenetic allometry combined. These sources of allometry were removed by using the residuals to examine variation within the sample. Furthermore, an additional regression of centroid size on age revealed a size effect of the XLHED mutation, such that XLHED individuals have a significantly smaller face than healthy controls (Control mean=0.009, XLHED mean=−0.0254, *P* = 0.003). XLHED and control individuals moderately differed from each other across PCs 1, 4, and 6. Together, PCs 1 through 6 accounted for 74% of the total shape variance. The first PC (32% of the total variance) captured shape variation concentrated in the nose and mouth (midfacial complex) and zygomatic region (Fig. 3A). Shape variance was also evident in the mandible, with the positive end of PC1 displaying a degree of mandibular prognathism, as deciphered from an anterior translation of midline landmarks 7 and 8 (Table 2). Compared to controls, individuals with XLHED displayed more protruding chin and mandible, high zygomatic arches, a narrower and more pointed nose, and a narrower mouth.

PC1 also separated XLHED and control individuals in terms of facial height. The XLHED shape described at the positive end of PC1 was an overall shorter face with relatively longer chin and shortened philtrum compared to control individuals, who scatter toward the negative end and zero of PC1. Furthermore, in comparison of the eigenvector lengths, we found strong association of landmarks located in the midface and upper face, and the mandible/chin in PCs 1–3 (Table S1). CVA of the XLHED cohort found no significant differences between mean facial shape according to ethnicity (Caucasian African American *P* = 0.3339; Hispanic African American *P* = 0.3456, Caucasian Hispanic *P* = 0.0568). These data indicate that the predominant shape effects observed result from XLHED, rather than ethnicity.

### Characteristic midfacial shape in XLHED individuals

Permutation tests (10,000 permutation rounds) using the Procrustes distance between groups defined by affected status revealed a significant midfacial shape difference between XLHED and control individuals (Procrustes distance = 0.0650, *P*<0.0001) with a characteristic midfacial shape in XLHED individuals. We then performed an additional permutation test, using the dataset with the three related individuals imputed into the Procrustes space. This analysis resulted in slightly but not significantly altered Procrustes distances (Procrustes distance = 0.0706 *P*<0.0001). Since including the other half of the sibling pairs did not appreciably alter the resultant shapes, all individuals were included in the final analyses due to the small sample size of the XLHED cohort. We performed a CVA using the unrelated subjects and projected the related individuals into this space using the CVA eigenvectors. CV1 showed that XLHED individuals had a relatively shorter face with a shortened philtrum and nasal columella, and displayed a degree of mandibular prognathism (Fig. 3B). XLHED individuals also had altered labium inferius oris shape, with a fuller and more rounded lower lip than controls. Narrower nasal ala and a more pointed nasal tip were also observed in XLHED individuals. We found no significant shape differences within the XLHED group when a permutation test was performed based on type of mutation (nonsense, missense, or deletion) or region of the EDA protein affected (TNF, furin, or transmembrane domain). Together, these findings show that individuals with XLHED have a characteristic craniofacial phenotype, statistically different from controls.

## Discussion

GM analysis on individuals with XLHED showed that, compared to control individuals, subjects with XLHED exhibit a quantitatively distinct set of craniofacial characteristics, including an overall reduction in size of the face, a shorter face, high zygomatic arches, relatively long chin, shortened philtrum, midface hypoplasia, fuller and more rounded lower lip, more protrusive chin and prognathic mandible, narrower and more pointed nose, and narrower mouth.

Previous reports that utilized anthropormorphic and cephalometric measurements have shown that male patients with XLHED exhibit decreased total facial height (Lexner et al. 2007), and this finding agrees with our study, in which XLHED individuals had relatively shorter and narrower facial shape than controls. Additionally, previous studies in XLHED individuals have reported the following: midfacial hypoplasia, with a retroclined nasal bone; short, retrognathic, anteriorly inclined maxilla; and a prognathic mandible, all of which are in agreement with our findings (Saksena and Bixler 1990; Johnson et al. 2002; Lexner et al. 2007).

Currently, cephalometric measurement of skeletal structures is the standard of care in orthodontics and oral surgery. Our study shows that 3D craniofacial morphometric analysis provides a more detailed, more efficient, and more accurate tool than 2D cephalometrics in diagnosis and treatment of individuals with XLHED and potentially other syndromes as well.

In addition, we attempted to determine phenotype–genotype correlations in the XLHED cohort based on 3D morphometric analysis. Overall, we found no significant differences in craniofacial structures based on type of *EDA1* mutation (nonsense, missense, or deletion) or region of the EDA protein affected (TNF, Furin, or transmembrane domain). This absence of genotype–phenotype correlation suggests that any mutations in *EDA* that cause significant loss of function can result in a similar craniofacial appearance, consistent with published reports that have not observed genotype–phenotype correlations in XLHED (Kobielak et al. 2001; Clauss et al. 2010; Zhang et al. 2011). Nevertheless, it remains possible that, with analysis of a larger cohort in the future, the utilization of 3D morphometrics might accurately distinguish subtle morphological variations that may highlight correspondingly subtle genotype–phenotype correlations.

Precise 3D craniofacial morphometric analysis thus is a powerful tool for rapid clinical diagnosis of XLHED, and may serve as a useful adjunct to genetic testing. In addition, the same technology may be applicable to diagnosis of female carriers of XLHED. Indeed, previous cephalometric analyses of female carriers of XLHED have reported a relatively short, retrognathic maxilla and retruded lips (Saksena and Bixler 1990). Thus, 3D craniofacial morphometric analysis is likely to become an important tool for the rapid identification of syndromes in the future. The ability to quantitatively define craniofacial phenotypes will improve the speed and accuracy of diagnosis, and as molecular therapies for conditions such as XLHED are developed, 3D morphometrics can help to pave the way for early identification and treatment.

## Supporting Information

**Figure S1.** Magnitude of shape change by principal component. Magnitude of shape change for PCs 1–3, as calculated from PC loadings. Magnitudes are magnified by 2×.

## References

[b1] Bhuiyan ZA, Klein M, Hammond P, van Haeringen A, Mannens MM, Van Berckelaer-Onnes I (2006). Genotype-phenotype correlations of 39 patients with Cornelia De Lange syndrome: the Dutch experience. J. Med. Genet.

[b2] Bookstein FL (1997). Morphometric tools for landmark data.

[b3] Cignoni P, Corsini M, Ranzuglia G (2008). MeshLab: an open-source 3D mesh processing system. ERCIM News.

[b4] Clauss F, Maniere MC, Obry F, Waltmann E, Hadj-Rabia S, Bodemer C (2008). Dento-craniofacial phenotypes and underlying molecular mechanisms in hypohidrotic ectodermal dysplasia (HED): a review. J. Dent. Res.

[b5] Clauss F, Chassaing N, Smahi A, Vincent MC, Clavas P, Molla M (2010). X-linked and autosomal recessive hypohidrotic ectodermal dysplasia: genotypic-dental phenotypic findings. Clin. Genet.

[b6] Courtney J, Blackburn J, Sharpe PT (2005). The ectodysplasin and NF*κ*B signalling pathways in odontogenesis. Arch. Oral Biol.

[b7] Hallgrimsson B, Jamniczky H, Young NM, Rolian C, Parsons TE, Boughner JC (2009). Deciphering the palimpsest: studying the relationship between morphological integration and phenotypic covariaton. Evol. Biol.

[b8] Hammond P, Hutton TJ, Allanson JE, Campbell LE, Hennekam RC, Holden S (2004). 3D analysis of facial morphology. Am. J. Med. Genet.

[b9] Hammond P, Suttie M, Hennekam RC, Allanson J, Shore EM, Kaplan FS (2012a). The face signature of fibrodysplasia ossificans progressiva. Am. J. Med. Genet.

[b10] Hammond P, Hannes F, Suttie M, Devriendt K, Vermeesch JR, Faravelli F (2012b). Fine-grained facial phenotype-genotype analysis in Wolf-Hirschhorn syndrome. Eur. J. Hum. Genet.

[b11] Heulens I, Suttie M, Postnov A, De Clerck N, Perrotta CS, Mattina T (2012). Craniofacial characteristics of fragile X syndrome in mouse and man. Eur. J. Hum. Genet.

[b12] Johnson EL, Roberts MW, Guckes AD, Bailey LJ, Phillips CL, Wright JT (2002). Analysis of craniofacial development in children with hypohidrotic ectodermal dysplasia. Am. J. Med. Genet.

[b13] Klingenberg CP (1998). Heterochrony and allometry: the analysis of evolutionary change in ontogeny. Biol. Rev. Camb. Philos. Soc.

[b14] Klingenberg CP (2011). MorphoJ: an integrated software package for geometric morphometrics. Mol. Ecol. Resour.

[b15] Kobielak K, Kobielak A, Roszkiewicz J, Wierzba J, Limon J, Trzeciak WH (2001). Mutations in the EDA gene in three unrelated families reveal no apparent correlation between phenotype and genotype in the patients with an X-linked anhidrotic ectodermal dysplasia. Am. J. Med. Genet.

[b16] Lexner MO, Bardow A, Bjorn-Jorgensen J, Hertz JM, Almer L, Kreiborg S (2007). Anthropometric and cephalometric measurements in X-linked hypohidrotic ectodermal dysplasia. Orthod. Craniofac. Res.

[b17] Mikkola ML (2009). Molecular aspects of hypohidrotic ectodermal dysplasia. Am. J. Med. Genet.

[b18] Montonen O, Ezer S, Saarialho-Kere UK, Herva R, Karjalainen-Lindsberg ML, Kaitila I (1998). The gene defective in anhidrotic ectodermal dysplasia is expressed in the developing epithelium, neuroectoderm, thymus, and bone. J. Histochem. Cytochem.

[b19] Rohlf FJ, Slice D (1990). Extensions of the Procrustes method for the optimal superimposition of landmarks. Syst. Biol.

[b20] Saksena SS, Bixler D (1990). Facial morphometrics in the identification of gene carriers of X-linked hypohidrotic ectodermal dysplasia. Am. J. Med. Genet.

[b21] Wiley DF, Amenta N, Alcantara DA, Ghosh D, Kil YJ, Delson E (2005). Evolutionary morphing. Proceedings of the IEEE Visualization 2005 (VIS '05).

[b22] Zelditch ML, Swiderski DL, Sheets DH, Fink WL (2004). Geometric morphometrics for biologists.

[b23] Zhang J, Han D, Song S, Wang Y, Zhao H, Pan S (2011). Correlation between the phenotypes and genotypes of X-linked hypohidrotic ectodermal dysplasia and non-syndromic hypodontia caused by ectodysplasin-A mutations. Eur. J. Med. Genet.

