# Supplementary figures and images for: Craniofacial morphometric analysis of individuals with X-linked hypohidrotic ectodermal dysplasia
Source: Mol Genet Genomic Med. 2014 May 20;2(5):422–9. doi: 10.1002/mgg3.84 (PMC4190877; doi:10.1002/mgg3.84)

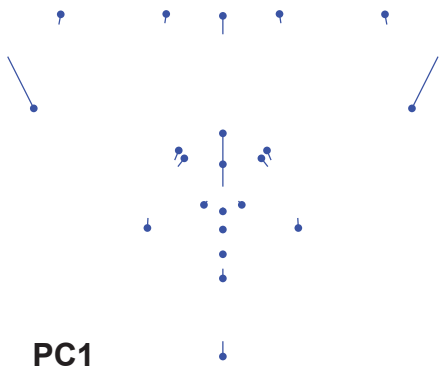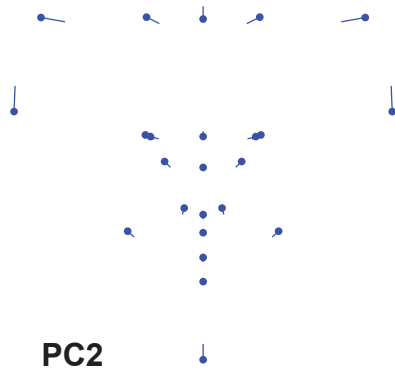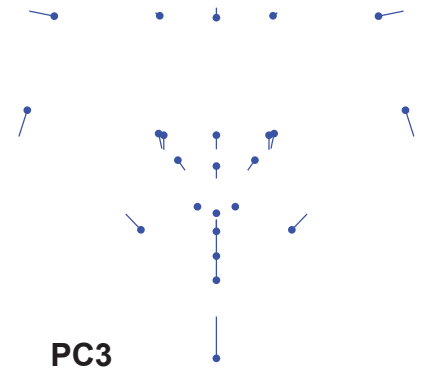

Supplement: Supplementary file 1 — Figure S1. Magnitude of shape change by principal component. Magnitude of shape change for PCs 1–3, as calculated from PC loadings. Magnitudes are magnified by 2×. [file mgg30002-0422-SD1.pdf]
