# Supplementary material for: Craniofacial morphometric analysis of individuals with X-linked hypohidrotic ectodermal dysplasia
Source: Mol Genet Genomic Med. 2014 May 20;2(5):422–9. doi: 10.1002/mgg3.84 (PMC4190877; doi:10.1002/mgg3.84)
Supplement: Supplementary file 2 — Figure S1. Magnitude of shape change by principal component. Magnitude of shape change for PCs 1–3, as calculated from PC loadings. Magnitudes are magnified by 2×. [file mgg30002-0422-SD2.docx]

**Supplemental Table 1:** Magnitude of shape change by landmark and principal component. Calculated from principal component loadings. Highlighted columns indicate the greatest five magnitudes for each PC. Loadings correspond to Supplementary Figure 1.

| **Landmark** | **PC1** | **PC2** | **PC3** |
| --- | --- | --- | --- |
| Naison | 0.143117456 | 0.116092848 | 0.119137884 |
| Pronasale | 0.241410529 | 0.15089658 | 0.310678676 |
| Subnasale | 0.171016368 | 0.193307205 | 0.143618644 |
| Labiale Superius | 0.02574881 | 0.196070283 | 0.074043264 |
| Stomion | 0.020125978 | 0.182290454 | 0.10970578 |
| Labiale Inferius | 0.049268372 | 0.101872805 | 0.280908153 |
| Sublabiale | 0.083130685 | 0.049530123 | 0.214015768 |
| Gnathion | 0.400064161 | 0.435640186 | 0.31867488 |
| Endocanthion | 0.113563547 | 0.17500632 | 0.037266746 |
| Exocanthion | 0.102852151 | 0.200614585 | 0.314381179 |
| Alare | 0.086500173 | 0.074737828 | 0.142510365 |
| Alare Curvature Point | 0.07772929 | 0.073955088 | 0.148630331 |
| Subalare | 0.088548816 | 0.148272502 | 0.171547156 |
| Christa Philtri | 0.03416358 | 0.185010957 | 0.06549604 |
| Chelion | 0.072636326 | 0.147697372 | 0.224723712 |
| Zygion | 0.523884875 | 0.352113085 | 0.23239031 |
